# NFYA1 Is Involved in Regulation of Postgermination Growth Arrest Under Salt Stress in *Arabidopsis*

**DOI:** 10.1371/journal.pone.0061289

**Published:** 2013-04-24

**Authors:** Yan-Jie Li, Yi Fang, Ya-Ru Fu, Jin-Guang Huang, Chang-Ai Wu, Cheng-Chao Zheng

**Affiliations:** State Key Laboratory of Crop Biology, Shandong Agricultural University, Taian, Shandong, People’s Republic of China; East Carolina University, United States of America

## Abstract

The nuclear factor Y (NF-Y), which is a ubiquitous transcription factor found in eukaryotes, is composed of three distinct subunits, namely, NF-YA, NF-YB, and NF-YC. Here, we firstly characterized the detailed function of the Arabidopsis NFYA1 factor. It is found that the *35S::AtNFYA1*-overexpressed lines were hypersensitive to salt stress and Abscisic acid (ABA) during the early-postgermination growth stages. The transgenic lines exhibited a severe postgermination growth arrest compared with the wild-type (WT) under salt stress and ABA treatment. Interestingly, sodium tungstate, which is an ABA synthesis inhibitor, restored the salt-sensitive phenotype of the *35S::AtNFYA1* lines. Results of the qRT-PCR analysis showed that the mRNA levels of *ABI3* and *ABI5*, as well as their downstream genes *AtEM1* and *AtEM6*, were more greatly upregulated under salt stress during seed germination in the transgenic lines compared with those in WT. On the other hand, the *NFYA1-RNAi* lines were found to be insensitive to salt stress and exhibited decreased levels of *ABI3*, *ABI5*, *EM1*, and *EM6* transcripts. Our results provide clear evidence supporting a role of *AtNFYA1* in regulating postgermination growth arrest under salt stress.

## Introduction

Seed germination is a critical process in the plant life cycle, which is determined by many environmental factors, such as temperature, light, oxygen, and water status, among others. Seeds often fail to germinate and develop under unfavorable conditions even after the break of dormancy. The Abscisic acid (ABA) is an important phytohormone that regulates the responses of plants to various environmental stresses, such as high salinity and drought [1]. ABA also plays a significant role in the mediation of seed dormancy, seed germination, and postgermination growth [2], [3]. ABA levels decrease upon seed inhibition to allow the seeds to germinate and develop into seedlings under normal conditions. However, ABA levels remain high under abiotic stress conditions, arresting plant growth and development [4]. The exposure of seeds to exogenous ABA during germination leads to a rapid but reversible arrest in postgermination growth. This ABA-mediated growth arrest of germinated seeds serves as a protective mechanism for the survival of young seedlings under stress conditions [2].

*ABI3* and *ABI5* are important ABA-insensitive genes that regulate the mediation of ABA-dependent growth arrest during germination [5], [6], [7], [8]. *ABI5* encodes a member of basic leucine zipper transcription factors, which can be induced by ABA only within a short poststratification interval, and thus defines a narrow developmental checkpoint following germination [8]. *ABI3* encodes a putative acidic domain transcription factor that is necessary for the expression of a large number of late embryogenesis genes, which are supposedly required for desiccation tolerance [7], [9]. The accumulation of *ABI3* is also triggered by ABA within a short developmental window. *ABI5* mRNA levels were found to be greatly reduced in *abi3-1* mutant. Additionally, *ABI5* can rescue the ABA-insensitivity of *abi3-1*. This phenomenon shows that *ABI3* acts upstream of *ABI5*, and therefore indicates the dependency of growth arrest efficiency on *ABI5* levels [8]. *AtEM1* and *AtEM6* are two important *Group 1 Late Embryogenesis Abundant* (*LEA*) genes that are expressed during the late period of embryo maturation and during the developmentally regulated period of dehydration at the end of seed development [10]. In the presence of ABA, *ABI5* can regulate the expression of *AtEM1* and *AtEM6* by binding to their promoters [8], [11]. Moreover, *AtEM1* and *AtEM6* can also be re-induced during the postgermination arrest process under water deficit while the *ABI5* expression is upregulated, which might be responsible for the acquired stress tolerance of germinated seeds [8].

Several studies revealed that *CPR5*, *WRKY2* and *HYL1* genes also mediate postgermination growth arrest. The phenotypes of *cpr5*, *wrky2* and *hyl1* mutants exhibited ABA-hypersensitivity during germination and displayed different degrees of postgermination growth arrest [12], [13], [14]. Moreover, these mutants showed significantly altered expression levels of *ABI3*, *ABI5*, *EM1*, *and EM6*, implying their direct or indirect involvement in the ABA-regulated postgermination pathways.

Nuclear factor Y (NF-Y) is a kind of ubiquitous transcription factor present in nearly all eukaryotes, which includes three subunits: NFYA, NFYB and NFYC [15], [16]. In animals, the NF-YB and NF-YC subunits firstly form a heterdimer in the cytoplasm, and then it is transported into the nucleus and recruits the NF-YA subunit to generate the mature, heterotrimeric NF-Y transcription factor [17], [18], [19]. This complex has high affinity and sequence specificity for the CCAAT box, which is a cis-element that exists in approximately 25% of eukaryotic gene promoters [18], [20], [21].

*Arabidopsis* has multiple genes encoding the three subunits, namely, 10 NF-YA, 13 NF-YB, and 13 NF-YC homologs [19]. Due to the potential combinatorial diversity among numerous NFY factors, as well as the high affinity and sequence specificity for the extensive CCAAT box in the eukaryotic genomes, these members play significant roles in the mediation of diverse genes and involve in regulating various processes. For instance, the Leafy Cotyledon1, (LEC1 or NFYB9) which was the first cloned and described plant NF-Y, is essential in both early and late embryo development and necessary for controlling the transition from embryo to adult status [22], [23], [24]. Altered expression of NFYB1 (HAP3a) and NFYB2 (HAP3b) in *Arabidopsis* could greatly affect the flowering time [21], [25], [26], Several NF-Y members also appear to be important in regulating drought responses, such as the maize NFYB1 and the Arabidopsis NFYA5, both of which could confer drought stress tolerance in the transgenic plants [27], [28],. Additionally, some NFYA subunits such as Arabidopsis NFYA2, NFYA3, NFYA5 were reported to participate in nitrogen nutrition [29], and broader functions of NFY factors like regulation of light signaling, ER stress, Chloroplast biogenesis *etc* were also revealed [29], [30].

Regarding Arabidopsis NFYA1, its possible role in delaying flowering was reported earlier under the control of a tissue-specific promoter [26]. Recently, Mu *etal* reported the function of NFYA1 in reproductive development and seed germination [31]. In this study, we demonstrate novel roles for NFYA1 in salt stress. Transgenic lines overexpressing NFYA1 were shown to be hypersensitive to salt stress during post-germination growth. Moreover, genes with known involvement in post-germination development, such as ABI3, ABI5, EM1, and EM6, were greatly upregulated in the transgenic lines. The results of this study show that NFYA1 can be an important regulator in the mediation of post-germination growth arrest under salt stress and ABA treatment.

## Materials and Methods

### Plant Material

*Arabidopsis thaliana* ecotype Columbia was used in this study. Surface sterilized seeds were sown on full strength MS medium supplemented with 3% sucrose and stratified at 4°C for 2 d in the dark prior to germination. Seedlings were grown on MS plates or soil under LD (16 h light/8 h dark) or SD (8 h light/16 h dark) conditions at 22°C. Seeds of *abi3, abi5* were obtained from the Arabidopsis Biological Resource Center (ABRC).

### Constructs and Generation of Transgenic Plants

To generate *35S::NFYA1* construct, *NFYA1* sequence without 3′UTR was PCR amplified from the *Arabidopsis* cDNA and inserted downstream of 35S promoter in the binary vector pBI121 after confirmed by sequencing. To generate *35S::NFYA1-GFP* construct, stop codon removed *NFYA1* was ligated in frame to GFP and confirmed by sequencing. To generate *AtNFYA1-RNAi* construct, the *Arabidopsis* pFGC5941 vector (ordered from ABRC) for dsRNA production was used. A 427 bp fragment of *NFYA1* cDNA was amplified by PCR using the forward primer 5′-TCTAGAGGCGCGCCGCTCTGCTGTGAATTTCCACT-3′ and the reverse primer 5′-GGATCCATTTAAATCCTGATATGGGTTTGGGACAC-3′. The fragment was first cloned between the AscI and SwaI sites of pFGC5941 before an inverted repeat of the same fragment was inserted into the *BamH*I and *Xba*I sites of pFGC5941 already containing the sense repeat. All the recombinant binary vectors were introduced into Agrobacterium tumefaciens strain GV3101 and the WT *Arabidopsis* were transformed by the floral dip method [32]. All the sequence information is from TAIR database (http://www.arabidopsis.org/). Details about other primer sequences are shown in Table S1.

### Microscopy Analysis

For DAPI staining, seedlings were stained in 0.2 mg/L DAPI for 15 min, washed three times in PBS solution. For GFP analysis, root tips from 3-day-old transgenic seedlings or onion epidermal cells carrying *35S::NFYA1-GFP* expression vector were mounted on slides, and then DAPI or GFP signal was observed with a fluorescence microscope (BX51, model 7.3; Olympus, Japan).

### Stress Assays

For germination assay under stresses, seeds were surface sterilized with 70% ethanol for 5 min, with 2.6% hypochlorite for 10 min, and then rinsed with sterile deionized water. Germination assays were carried out with three replicates of 50 seeds. Seeds were sown on MS medium supplemented with 3% sucrose, and the plates were placed at 4°C for 3d in the dark and then transferred to the growth chamber (16 h light/8 h dark) at 22°C. The seeds were regarded as germinated when the radicles protrude from the seed coat. For direct comparison of germination rates, each plate was subdivided, and seeds of all genotypes were put on the same plate.

### Statistical Analysis

Data were subjected to Data Processing System (DPS) and significant differences between individual means established using a Student’s t test. Differences at the 1% level were considered significant and denoted by the lowercase letters among different groups.

### Quantitative RT-PCR

For qRT-PCR, Reverse transcription reactions were performed using 5 µg RNA by M-MLV reverse transcriptase (Transgene, CHINA) according to the supplier’s manual after incubation with RNase-free DNase I. QRT-PCR reactions were performed with a Bio-Rad real-time thermal cycling system using SYBR-Green to detect gene expression abundances. The reaction mixture (25 µl) contained 0.5 µM of each primer and appropriate amounts of enzymes, cDNA and fluorescent dyes. All runs used a negative control without adding target cDNA, resulting in no detectable fluorescence signal from the reaction. A range of five dilutions of the total cDNA was tested in the same conditions as the samples. Amplification reactions were initiated with a pre-denaturing step at 95°C for 30s and followed by denaturing (95°C for 5s), annealing (60°C for 10s) and extension (72°C for 15s) steps for 49 cycles during the second stage, and a final stage of 55°C to 95°C to determine dissociation curves of the amplified products. All reactions were done in at least three replicates. Data were analyzed using Bio-Rad CFX Manager software. Primer information for qRT-PCR assay is included in Table S1.

## Results

### Characterization of the *AtNFYA1* Transcription Factor Expression

**Figure 1:**
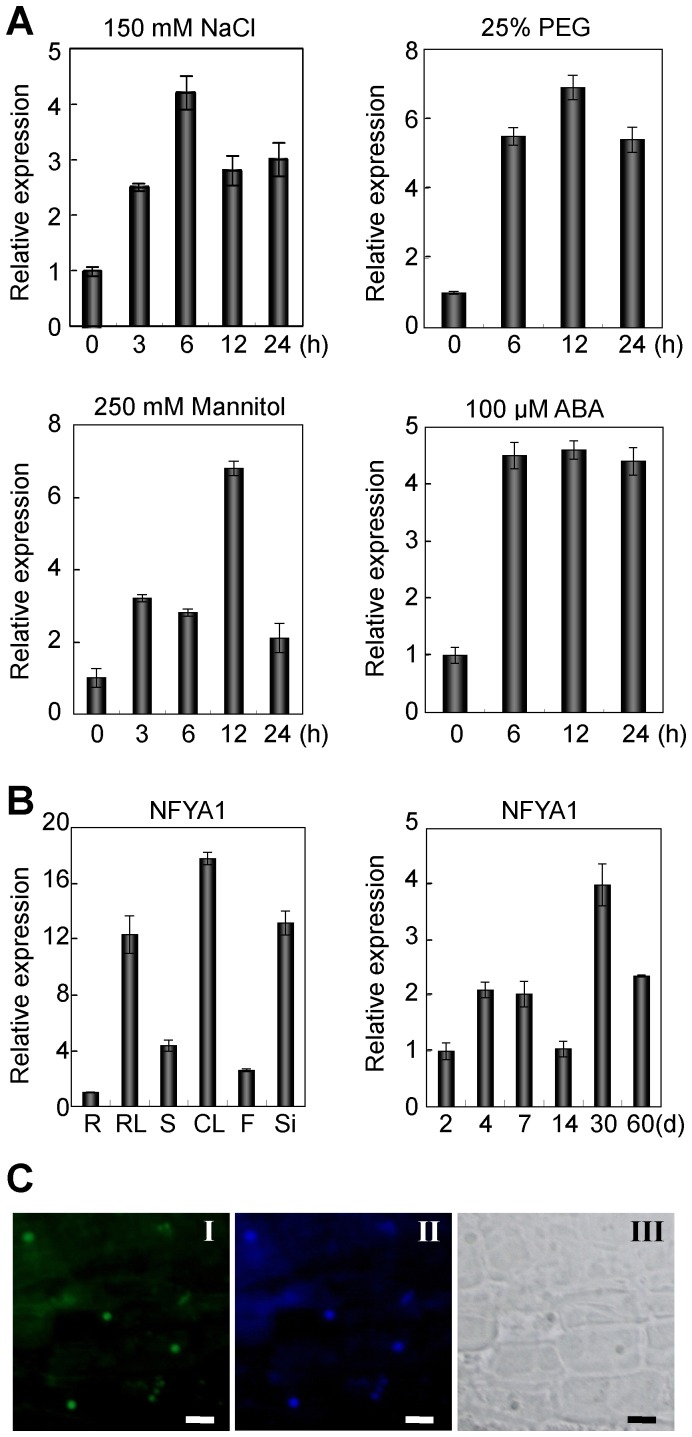
*AtNFYA1* expression pattern and transcriptional regulation. (A) Induction of *AtNFYA1* by NaCl, PEG, Mannitol and ABA assessed by qRT-PCR. 2-week-old *Arabidopsis* wild type seedlings growing on agar medium under LD condition were treated with the hydroponic solution in petri dishes with filter paper. Expression levels of non-treated samples (0 h) were set to 1.0. (B) Spatio-temporal expression of *AtNFYA1* assessed by qRT-PCR. *NFYA1* accumulation in roots and 2-day-old seedlings was set to 1.0, respectively. R: roots, RL: rosette leaves, S: stems, CL: cauline leaves, F; flowers, Si: siliques. qRT-PCR quantifications were normalized to ACTIN. Error bars represent SE for three independent experiments. (C) Nuclear localization of *NFYA1*. The *NFYA1-GFP* fusion construct was expressed in transgenic *Arabidopsis* under the control of the CaMV 35S promoter, and GFP signal was observed in the nucleus of the root tip cells (I), with DAPI staining of the nucleus (II) for comparison and morphology of the cells under bright field (III). Bar, (I)–(III), 100 µm.

To explore the possible biological roles of the NFYA family under abiotic stress, gene analysis was firstly performed by Genevestigator database (www.genevestigator.com), and the *NFYA1* (At5g12840) was found to respond to various environmental stimuli. Subsequently, qRT-PCR analysis was performed, and the results indicated that the expression of *NFYA1* was obviously induced by NaCl, mannitol, PEG, and ABA treatments (Fig. 1A), suggesting that *NFYA1* is involved in plant responses to environmental stimuli. In addition, the spatio-temporal expression pattern shows that the *Arabidopsis NFYA1* can be detected in all developmental stages from 2-day-old to 60-day-old plants, and it is preferentially expressed in leaves and siliques, while weakly expressed in roots and flowers (Fig. 1B).

The NFYA factors in mammals are located in the nucleus and interact with the NFYB/NFYC dimers transported from the cytoplasm [16]. In this study, a C-terminal fusion to the green fluorescent protein (GFP) was generated under the control of the cauliflower mosaic virus 35S promoter to confirm the subcellular localization of the NFYA1 protein. The GFP signal in the *35S::NFYA1-GFP* transgenic *Arabidopsis* was observed predominantly in the nucleus of the root tip cells (Fig. 1C). In addition, the nuclear localization was also confirmed in onion epidermal cells via transient expression (Fig. S1).

### Overexpression of *AtNFYA1* Leads to Postgermination Growth Arrest under Salt Stress

**Figure 2:**
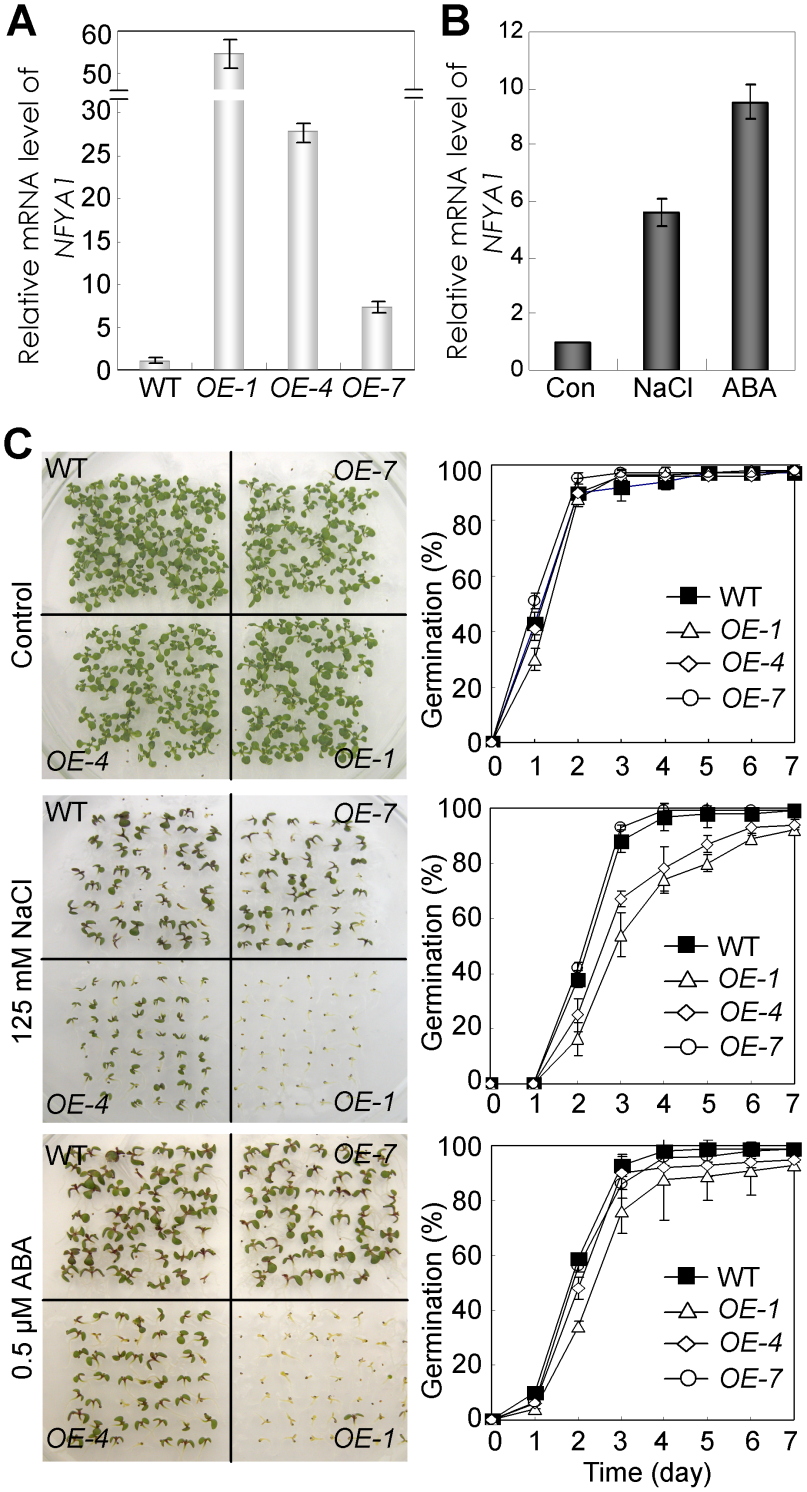
*35S::NFYA1* transgenic lines are hypersensitive to salt stress and ABA during postgermination growth. (A) Relative expression levels of different *35S::NFYA1* lines assessed by qRT-PCR. 2-week-old *Arabidopsis* seedlings on MS agar medium under LD condition were harvested and RNA was extracted. Expression levels of *NFYA1* in wild type were set to 1.0. (B) Accumulation of *NFYA1* mRNA in germinated seeds of under ABA and salt treatment. Wild type seeds were germinated on MS liquid medium moistened filter paper for 24 hours after stratification, and then were transferred onto filtered paper moistened with water (control), 125 mM NaCl and 0.5 µM ABA respectively. After treated for 8 hours, the samples were harvested and RNA was extracted. Here, three biological repeats were done and each qRT-PCR was also performed three times. (C) Wild type, *NFYA1* transgenic lines *OE-1*, *OE-4* and *OE-7* growing on MS medium supplemented with 0 mM (control) and 125 mM NaCl for 14 days, and germination rate of WT, *OE-1*, *OE-4* and *OE-7* seeds on the above medium counted for 7 days after stratification. For these data, at least three independent biological repeats were done, and above 50 seeds were used for each line. Pictures were taken 14 d after stratification.

To demonstrate the function of *NFYA1* in the regulation of plant responses to abiotic stresses, the transgenic plants overexpressing *NFYA1* under the control of the CaMV 35S promoter were generated. Several independent lines that express various levels of *NFYA1* were obtained. Line 1 (*OE-1*), Line 4 (*OE-4*), and Line 7 (*OE-7*), which exhibited high, moderate, and low *NFYA1* mRNA levels, respectively, were chosen for further analysis (Fig. 2A). Although NFYA1 mRNA is obviously induced by salt and ABA in both seedlings (Fig. 1A ) and germinated seeds (Fig. 2B ), and the *NFYA1* transgenic plants were observed to be clearly tolerant to salt stress (Fig. S2), when considering the seed germination and subsequent growth of WT and *NFYA1* transgenic seedlings, a remarkable postgermination growth arrest of *OE-1* was observed after seed germination (Fig. 2C). As shown in Fig. 2C, the WT and *NFYA1* transgenic plants showed no difference after growing for 2 weeks on the medium without NaCl. However, on the medium supplemented with 125 mM NaCl, *OE-1* germinated but failed to develop into seedlings, and *OE-4* exhibited smaller seedlings as well as weaker growth compared with WT and *OE-7*. These observations indicate that the sensitivity of the germinated seeds to salt stress depends on the *NFYA1* expression levels. Additionally, when we grew the same transgenic lines on medium supplemented with ABA, postgermination growth arrest was also observed, and a more severe phenotype was found in the *OE-1* plants than the other two weaker lines *OE-4* and *OE-7*.

### *AtNFYA1* Functions in the Postgermination Checkpoint

**Figure 3:**
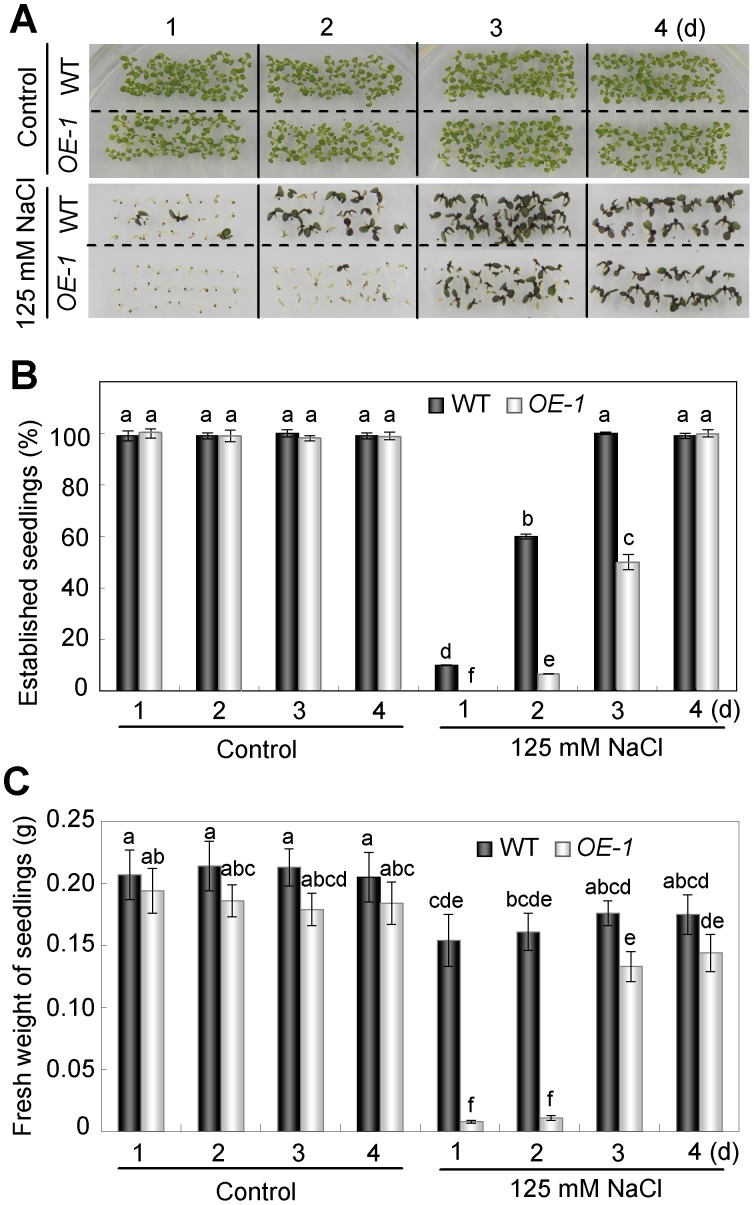
*35S::NFYA1* transgenic lines are hypersensitive to salt stress during a limited developmental window. (A) Seeds of WT and *OE-1* lines were sown on MS medium for germination, and after 1d, 2d, 3d and 4d, the germinated seeds were transferred respectively onto MS medium supplemented with 125 mM NaCl for postgermination growth for 14 days under LD condition. (B) The number of established seedlings under each condition was counted. (C) After 14 days, the fresh weight of 50 transferred seedlings under each condition was measured. For these data, at least three independent biological repeats were done. Samples with different letters are significantly different: P<0.01.

Since we saw clearly salt tolerance in the *NFYA1*-overexpressed seedlings and big plants (Fig. S2), whereas severe growth quiescence of the transgenic lines after seed germination, we assume that *NFYA1* functions in regulating the postgermination growth arrest of the plants during a very short period after seed germination, To figure out the developmental stage that *NFYA1* might involve in, the seeds of WT and *OE-1* were geminated under normal conditions for 1, 2, 3, and 4 days, respectively, after 3-day stratification at 4°C. Then, the germinated seeds (or young seedlings) were transferred onto MS medium supplemented with 125 mM NaCl and exposed to salt stress for two weeks. As shown in Fig. 3, the proportion of green seedlings gradually increased as the delay of the transfer (Fig. 3B). The growth arrest happened to almost all transgenic seedlings transferred from normal condition to salt stress medium within 2 days after germination. They showed nearly no increase of fresh weight and remained white and quiescent after 2 weeks under salt stress condition. (Fig. 3C). However, the exposure of the 3- and 4-day-old young transgenic seedlings growing on MS medium to salt stress condition failed to arrest their subsequent growth (Fig. 3B,C). Therefore, *NFYA1* is hypothesized to be involved in the mediation of postgermination growth arrest under salt stress during a limited developmental window before the seedlings turn green.

### Involvement of *AtNFYA1* in the ABA Signaling Pathway Instead of in the Synthesis Pathway

**Figure 4:**
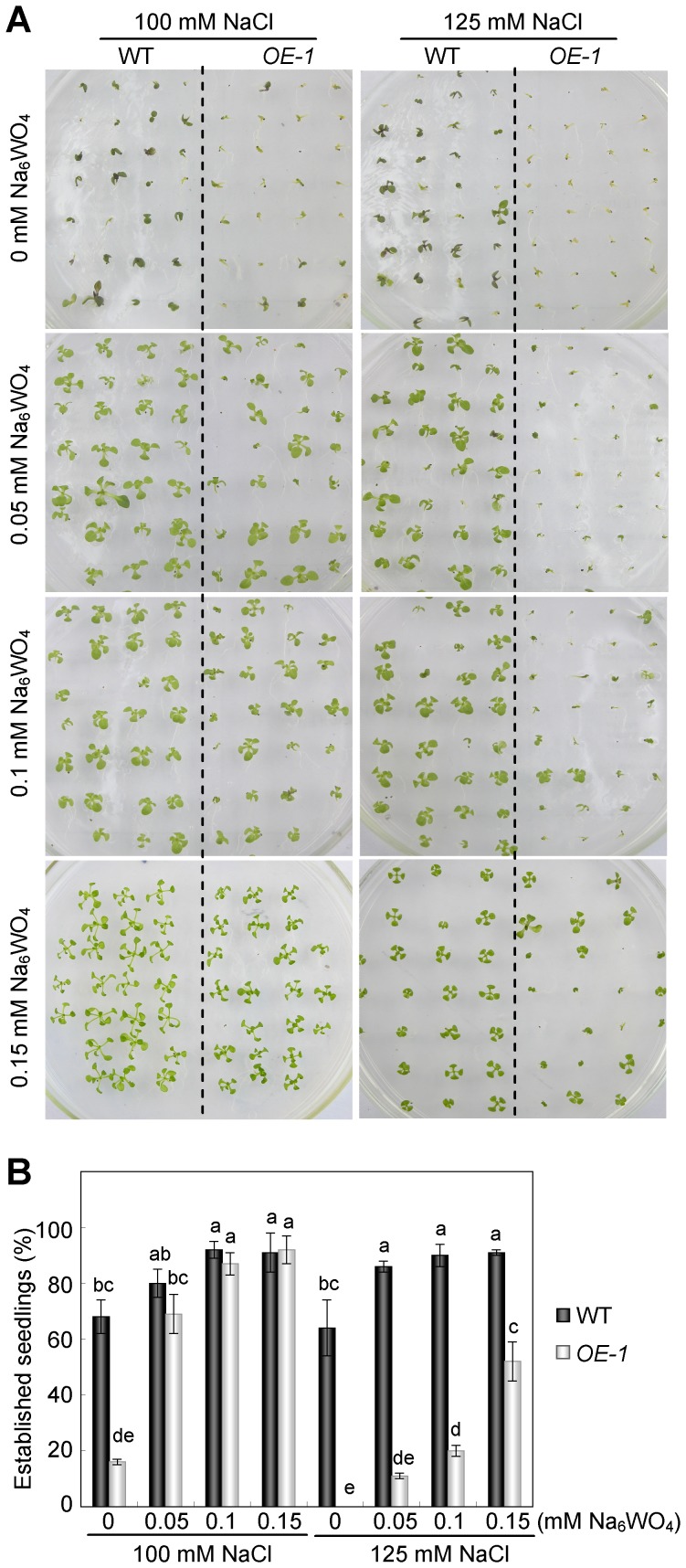
Sodium tungstate recovered the postgermination growth arrest of *NFYA1* overexpression line under salt stress. (A) WT and *35S::NFYA1* seeds were germinated on MS medium for 24 hours after stratification and transferred onto MS medium supplemented with NaCl and Na_6_Wo_4_ of different concentrations. (B) The rate of established seedlings was counted after growing for 14 days on NaCl and sodium tungstate medium. Samples with different letters are significantly different: P<0.01.

ABA is a critical signaling molecule involved in seed germination, postgermination growth, and abiotic stress resistance. In this study, to reveal whether the postgermination growth arrest under salt stress affected the ABA-related pathway. Sodium tungstate (Na_6_Wo_4_), which is one of the ABA synthesis inhibitors, was employed to block the endogenous ABA synthesis under salt treatment [33], [34], [35], [36]. As can be seen in Fig. 4, the application of sodium tungstate led to better growth of both the WT and *OE-1* plants under salinity conditions and recovered the growth arrest of *OE-1* seeds. Under more severe stress, higher concentration of sodium tungstate is required for the recovery of the salt sensitive phenotype (Fig. 4). For instance, the development of most *OE-1* seeds remained quiescent on MS medium supplemented with 125 mM NaCl and 0.1 mM sodium tungstate. However, in the presence of 0.15 mM sodium tungstate, they began to turn green and exhibit normal growth, implying that sodium tungstate has a dose effect in recovering the arrested growth of the *NFYA1* transgenic seedlings.

**Figure 5:**
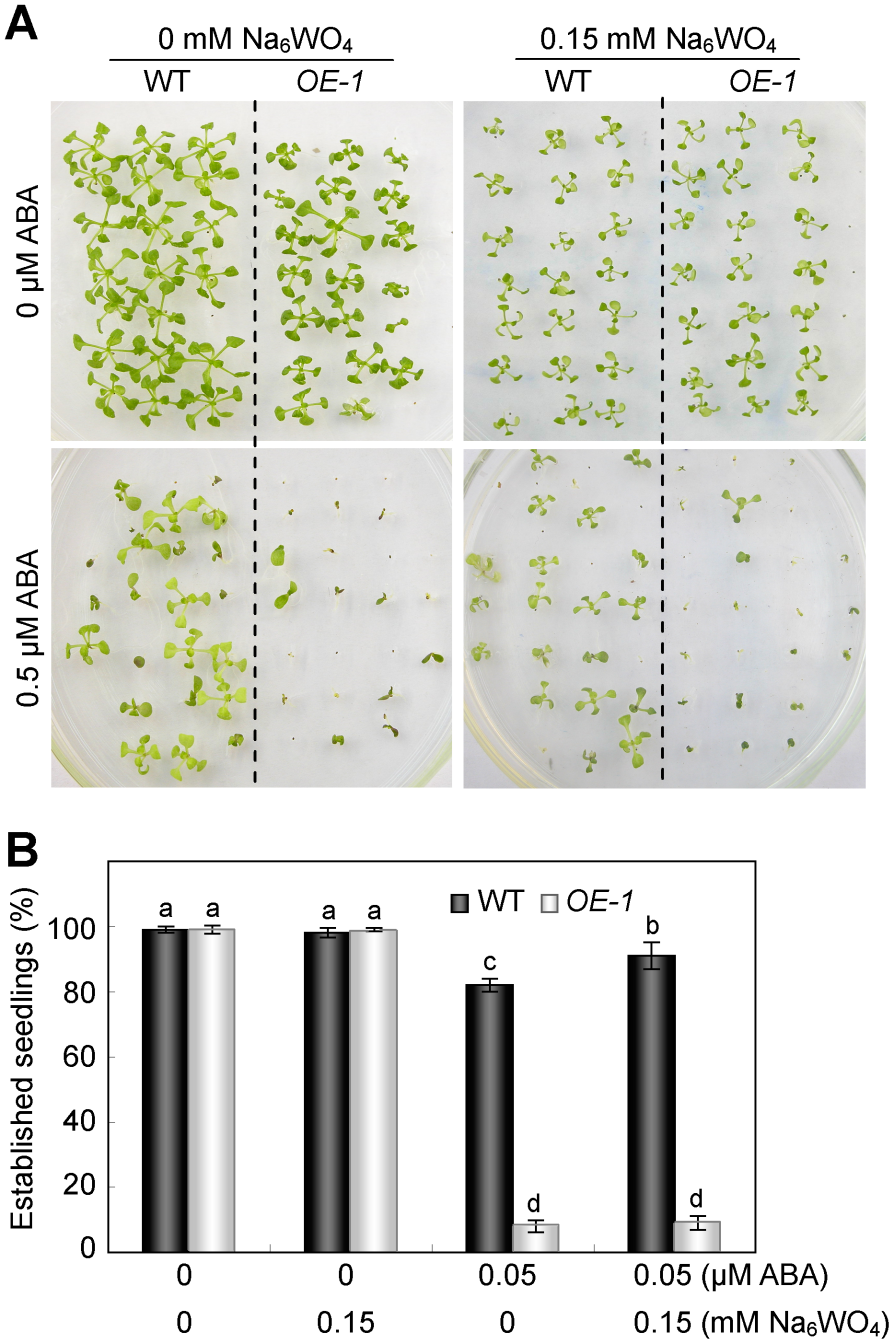
Sodium tungstate cannot recover the postgermination growth arrest of *NFYA1* overexpression line under exogenous ABA treatment. (A) WT and *35S::NFYA1* seeds were germinated on MS medium for 24 hours after stratification and transferred onto MS medium supplemented with ABA and Na_6_Wo_4_ of different concentrations. (B) The rate of established seedlings was counted after 14 days growing on the medium under LD condition. Samples with different letters are significantly different: P<0.01.

However, the concurrent application of exogenous ABA with sodium tungstate to the WT and *OE-1* plants failed to recover the quiescent transgenic seedlings to normal development. In addition, sodium tungstate alone cannot lead to any difference in between the WT and *35S::NFYA1* transgenic lines, which could serve as a control showing that sodium tungstate contributes nothing to the arrested growth of the germinated seeds (Fig. 5). These findings reasonably demonstrate that the *NFYA1* participates in regulating the ABA signaling pathway rather than the ABA synthesis pathway.

### *NFYA1-RNAi* Line is Insensitive to Salt Stress during Postgermination Growth

**Figure 6:**
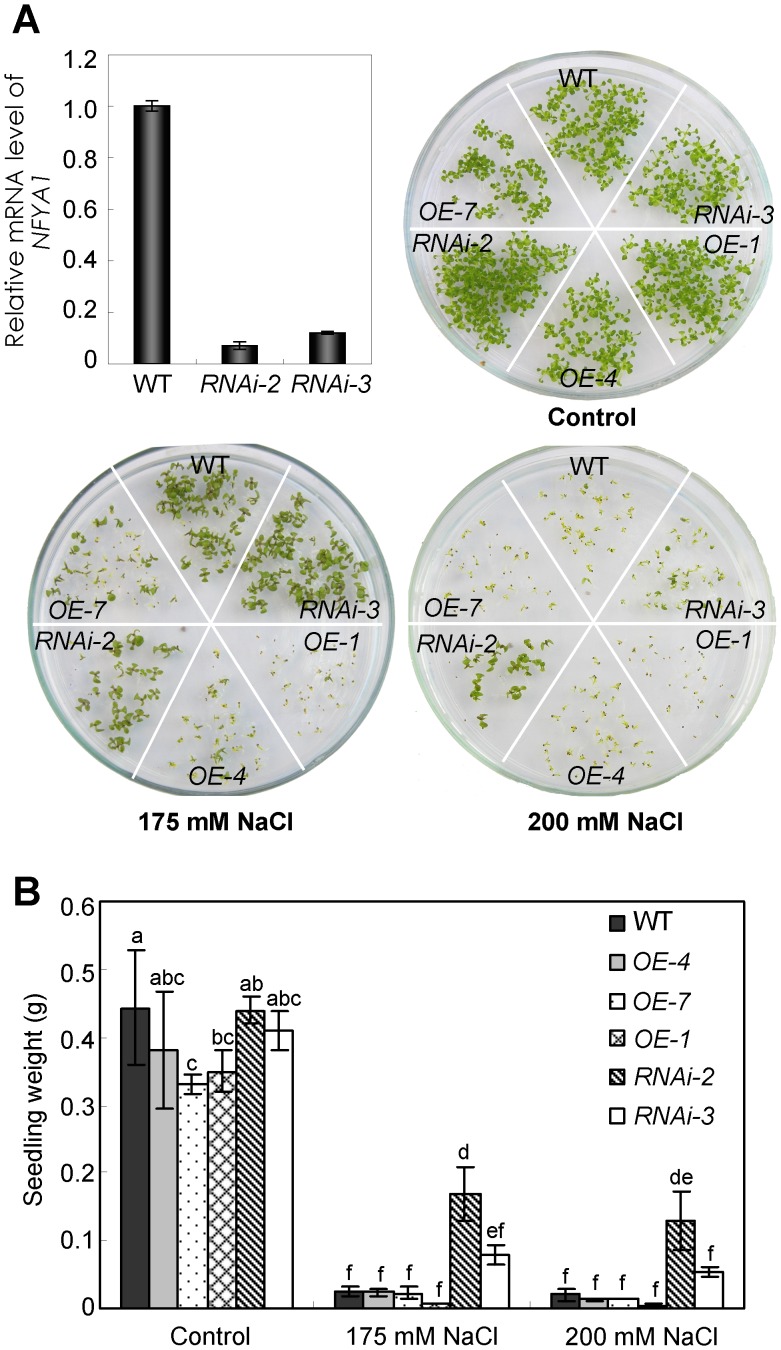
*NFYA1-RNAi* lines are tolerant to salt stress during germination and postgermination growth. (A) Expression level of *NFYA1* in *NFYA1-RNAi* lines assessed by qRT-PCR. Phenotype of wild type, *NFYA1-RNAi* lines and *35S::NFYA1* lines growing on MS medium supplemented with 0 mM (Control), 175 mM and 200 mM NaCl for 14 days under LD condition, and then weights of 25 seedlings were measured for each line (B). Three replicates were performed for each experiment. Samples with different letters are significantly different: P<0.01.

To further demonstrate the role of NFYA1 in regulating postgermination growth, we generated NFYA1-RNAi lines driven by 35S promoter. RNAi-2 and RNAi-3, in which the NFYA1 mRNA level was dramatically downregulated, were chosen for further experiment (Fig. 6A). As shown in Fig. 6B, both RNAi-2 and RNAi-3 could develop into green seedlings under 175 mM NaCl like WT, while the NFYA1 overexpression lines failed. Under higher salt stress (200 mM NaCl), where the growth arrest also happened to WT, the RNAi lines remain growing. These results indicate that NFYA1 acts as a negative regulator in affecting the seed postgermination growth, which is consistent with our above results.

### *AtNFYA1* Affects the ABA Signaling Pathway as an Upstream Regulator

**Figure 7:**
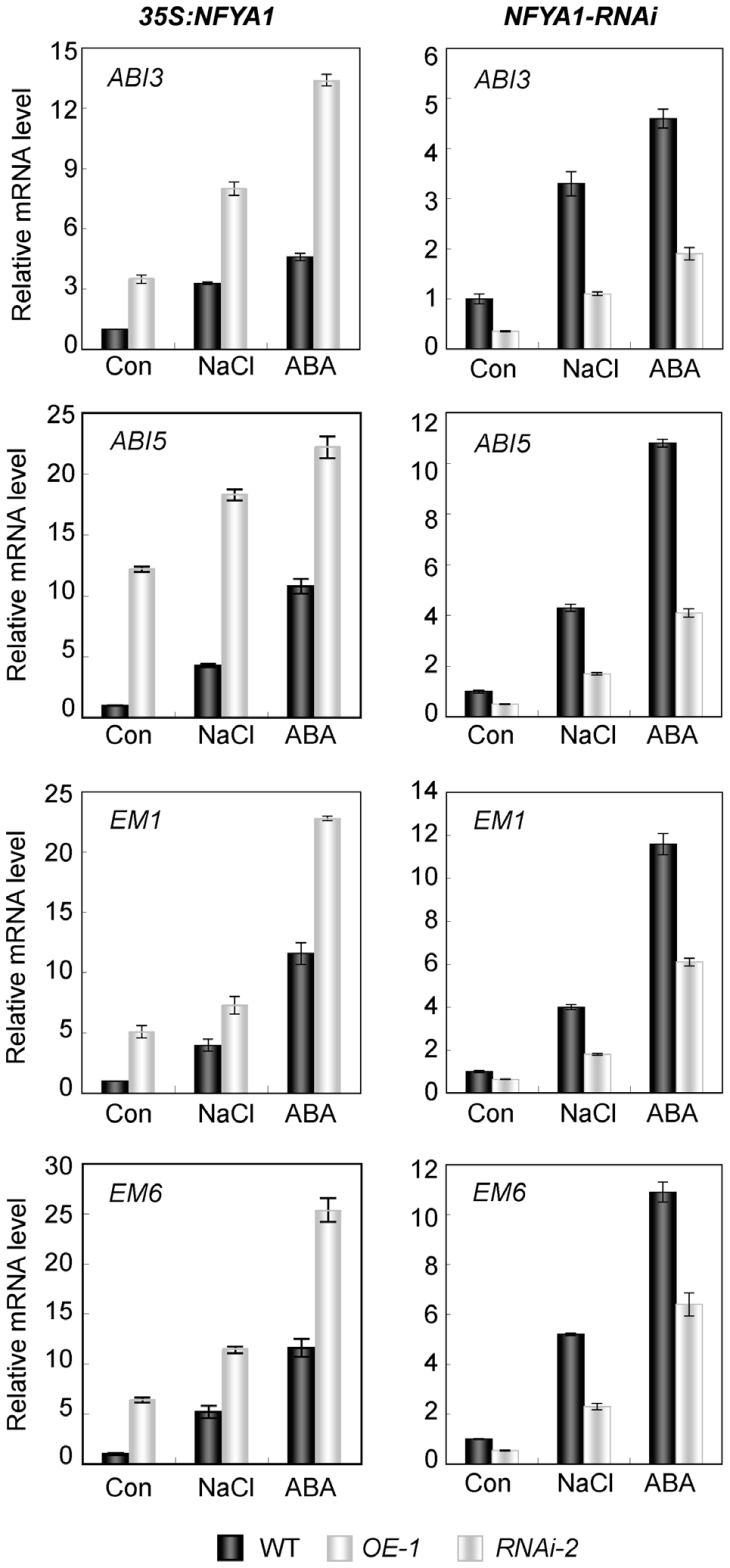
Expression levels of ABA pathway-related genes in *35S::NFYA1* line (OE-1) and *NFYA1-RNAi* transgenic line (*RNAi-2*) detected by qRT-PCR. Seeds were germinated on MS liquid medium moistened filter paper for 24 hours after stratification, and then were transferred onto filtered paper moistened with water (Con), 125 mM NaCl and 0.5 µM ABA respectively. After treated for 8 hours, the samples were harvested and RNA was extracted. Here, three biological repeats were done and each qRT-PCR was also performed three times.

The aforementioned study indicated that the growth arrest of the germinated seeds under salt stress is an adaptive mechanism regulated by the ABA signaling pathway. To determine the variety of genes affected and which one(s) contributed to the growth arrest of the seeds within this short developmental period, the *ABI3* and *ABI5* expressions were detected using qRT-PCR in the germinated seeds of WT and *NFYA1* transgenic lines treated with 125 mM NaCl and 0.5 µM ABA. Compared with WT, both *ABI3* and *ABI5* were elevated in the *NFYA1* transgenic lines under normal conditions, demonstrating that *NFYA1* acted upstream and positively regulated the *ABI3* and *ABI5* expressions. However, compared with normal condition (Control), higher expression levels of *ABI3* and *ABI5* were observed in the *NFYA1* transgenic lines under NaCl and ABA treatments. In addition, *EM1* and *EM6*, which act downstream of *ABI5*, exhibited similar expression patterns as those of *ABI3* and *ABI5* under these conditions (Fig. 7). The expressions of other genes involved in the seed germination and the ABA signaling pathway, such as *ABI3-interacting protein 3* (*AIP3*), *ABI4*, and *RAB18*, were also assessed, but only minor expressional changes were observed in the WT and *NFYA1* transgenic lines (data not shown). However, in the *NFYA1-RNAi* lines, the expression levels of *ABI3*, *ABI5*, *EM1*, and *EM6* were dramatically downregulated (Fig. 7). The above data suggested that *NFYA1* is involved in regulating ABI3-regulated cascades of the ABA signaling pathway.

**Figure 8:**
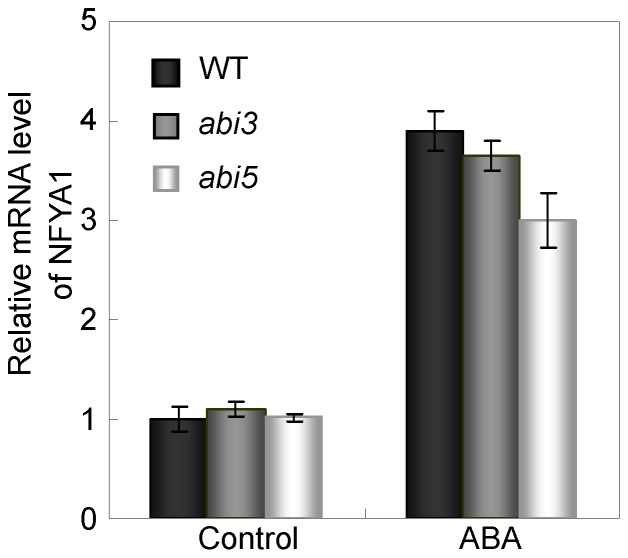
Expression of *NFYA1* in WT and *abi3, abi5* mutants detected by qRT-PCR. Seeds of WT, *abi3, abi5* were germinated on MS liquid medium moistened filter paper for 24 hours after stratification, and then were transferred onto filtered paper moistened with water (control), 125 mM NaCl and 0.5 µM ABA, respectively. After treated for 8 hours, the samples were harvested and RNA was extracted. Here, three biological repeats were done and each qRT-PCR was also performed three times.

To further confirm the above results, we detected the expression of NFYA1 in the *abi3* and *abi5* knockout mutants. The qRT-PCR results revealed that the level of *NFYA1* is not altered in both mutants under control conditions, and it is increased to similar levels in WT and the mutants under ABA treatments (Fig. 8). These findings demonstrate that ABA induced expression of *NFYA1* is not dependent on *ABI3* and *ABI5*, which is supportive to our conclusion that *NFYA1* acts upstream of *ABI3* and *ABI5* to regulate postgermination growth.

## Discussion

*Arabidopsis NFYA1* is a member of the NFY family, whose function is to be discovered. In our study, we reported a novel role of *AtNFYA1* in mediating seed postgermination growth arrest under salt stress, which also affected the ABA signaling pathway. It is observed that the *NFYA1*-overexpressed lines are hypersensitive to salt treatment in a does-dependent manner during seed postgermination growth. Dramatic development quiescence of the young embryos was observed in the presence of NaCl (Fig. 2C). It is, however, inconsistent with our initial finding that *NFYA1* is remarkably induced by several abiotic stresses such as salt, mannitol and PEG (Fig. 1), and the *35S::NFYA1* plants exhibit a salt resistant phenotype compared with that of wild type *Arabidopsis* (Fig. S2). These interesting observations demonstrate that *NFYA1* is involved in different signaling pathways at different developmental stages.

Under salt stress, it is found that the hypersensitivity of *NFYA1* overexpressers only confined in a limited developmental window during the postgermination growth, which is, before the cotyledons turn green and develop into young seedlings (Fig. 3). It is a very important checkpoint in this period under salt stress, because postgermination growth arrest can protect the germinated seeds from water deficit [2]. It is documented that the application of exogenous ABA can lead to arrested growth of the developing embryos after germination, and the formation of quiescent, germinated seeds is considered an adaptive mechanism to increase the survival rate of young seedlings under stresses [2], [37]. Genes involved in the regulation of postgermination growth arrest was also revealed in other studies by mutant screening, such as *cpr5*, *wrky2* and *hyl1*, and most of the knockout lines were dependent on the ABA signaling pathways and hypersensitive to exogenous ABA [12], [13], [14]. Several abiotic stresses, particularly water stress, can result in the accumulation of endogenous ABA, which in turn, triggers the upregulation of the defense system in plants to abiotic stresses [35], [38]. Apart from NaCl (Fig. 2C), ABA was also identified as an efficient stimulus of postgermination growth arrest in this study (Fig. 2C). Further experiments revealed that the salt-sensitive phenotype of the *35S::NFYA1* lines can be relieved by the application of the ABA synthesis inhibitor sodium tungstate, suggesting that salt stress might lead to the overaccumulation of endogenous ABA in the *NFYA1* transgenic seeds (Fig. 4). However, sodium tungstate failed to recover the arrested seeds into normal growth under exogenous ABA treatment (Fig. 5), implying that the growth arrest of the germinated seeds is not due to the increase of ABA but as a result of the perturbation of the downstream ABA signaling pathway. These findings further demonstrate that the *Arabidopsis NFYA1* participates in regulating the ABA signaling pathway rather than the ABA synthesis pathway.

The transcription factors *ABI3* and *ABI5* are known to be important regulators of ABA-dependent growth arrest during seed germination and early growth, and their expressions define a narrow developmental checkpoint following germination. *ABI5* acts downstream of *ABI3*, and ABA-induced *ABI5* can activate the expression of two late embryogenesis-abundant genes *EM1* and *EM6* by occupying their promoters [8], [11]. To gain insight into the *NFYA1* function during seed early growth after germination, we analyzed the expression levels of the above four genes in WT and *35S::NFYA1* transgenic lines. Interestingly, *ABI3*, *ABI5*, and their downstream genes *EM1* and *EM6* were all dramatically elevated in *NFYA1*-overexpressed lines and were downregulated in *NFYA1-RNAi* lines under ABA treatment and salt stress (Fig. 7). As compared with WT *Arabidopsis*, the higher expression levels of the four genes in *NFYA1*-overexpressed lines stimulated growth arrest. Here, our study reported that *NFYA1* might represent another branch in regulating the seed postgermination growth arrest via the *ABI3*-controlled cascades.

Some individual NFY subunits were reported to play pleiotropic regulating roles during plant growth and development. In our study, we observed novel functions of NFYA1 in abiotic stress responses at different developmental stages. Recently, Mu *etal*. reported the involvement of NFYA1 in male gametogenesis, embryogenesis and seed development [31]. These pleiotropic functions of *NFYA1* might be explained by the diverse combination of NFYA1 with other NFYB/NFYC factors, which thus affected different regulating pathways. In a word, it is interesting to gain insight into the connection of distinct NFYA1 functions, and further study on protein-protein or protein-DNA interactions is also needed to investigate the inner mechanisms.

## Supporting Information

Figure S1 **Specific localization of NFYA1 in the nucleus of onion epidermal cells.** Agrobacterium tumefaciens strain LBA4404 carrying 35S::GFP and *35S::NFYA1-GFP* expression vectors was transformed into the onion epidermal cells and then visualized with a fluorescence microscope (BX51, model 7.3; Olympus, Japan). Bar, 100 µm.
(TIF)

Figure S2 **Enhanced salt stress tolerance of** ***35S:NFYA1*** **transgenic plants.** (A) WT, *OE-1* and *OE-4* seeds were germinated on MS medium and the 5-day-old seedlings were transferred onto MS control medium without NaCl and MS medium supplemented with 125 mM NaCl for 10 days, (B) Statistical analysis of root elongation, (C) salt stress tolerance and (D) survival rate of 4-week-old WT and *OE-1* plants under SD conditions after treated with 0 mM (Control) and 250 mM NaCl for 10 days. Samples with different letters are significantly different: P<0.01.
(TIF)

Table S1 **Primers used in the qRT-PCR assay.**
(TIF)
